# Low rate of gut colonization by extended-spectrum β-lactamase producing Enterobacteriaceae in HIV infected persons as compared to healthy individuals in Nepal

**DOI:** 10.1371/journal.pone.0212042

**Published:** 2019-02-19

**Authors:** Supram Hosuru Subramanya, Indira Bairy, Niranjan Nayak, Shashiraja Padukone, Brijesh Sathian, Shishir Gokhale

**Affiliations:** 1 Manipal College of Medical Sciences, Pokhara, Nepal; 2 Melaka Manipal Medical College, Manipal Academy of Higher Education, Manipal, India; 3 Jawaharlal Institute of Postgraduate Medical Education & Research, Puducherry, India; University of Georgia, UNITED STATES

## Abstract

A worldwide increase in the gastrointestinal colonization by extended-spectrum β-lactamase (ESBL)-producing bacteria has been observed. Their prevalence amongst Healthy People Living with HIV (HPLWH) has not been investigated adequately. The aim of this study was to determine and compare the rates of and risk factors for intestinal carriage and acquisition of extended-spectrum β-lactamase producing Enterobacteriaceae (ESBL-E) and carbapenemase-producing Enterobacteriaceae (CPE) among healthy people living with HIV (HPLWH) and healthy HIV negative population in the community. A cross-sectional study was conducted. Rectal swabs from HPLWH (n = 119) and HIV negative individuals (n = 357) from the community were screened for ESBL and CPE. Phenotypically confirmed ESBL-E strains were genotyped by multiplex PCR. The risk factors associated with ESBL-E colonization were analyzed by a multivariable conditional logistic regression analysis. Specimen from 357 healthy volunteers (213 female and 144 male) and 119 HPLWH (82 female and 37 male) with a median age of 30 [IQR 11–50] years were included in the study. ESBL colonization were found in 45 (37.82% [CI 29.09, 47.16]) and 246 (68.91% [CI 63.93, 73.49]), HPLWH and healthy HIV negative participants respectively. HPLWH had lower ESBL carriage rate (odds ratio 0.274 [CI 0.178, 0.423]) compared to healthy HIV negative subject’s (p<0.01). In this study, no carbapenemase-producing bacteria were isolated.CTX-M-15 type was the most predominant genotype in both groups. Livestock contact and over-the-counter medications were significantly associated with a higher ESBL-E carriage rate among healthy subjects. This is the first study in Nepal that has demonstrated a high rate of gut colonization by ESBL-E in the community, predominantly of blaCTX-M-15 genotype. This study divulges the low fecal carriage rate of ESBL producing bacteria in HPLWH group compared to healthy individuals in western Nepal. The factors responsible for this inverse relationship of HIV status and gut colonization by ESBL-E are unidentified and require further large-scale study.

## Introduction

Antimicrobial resistance has become a serious threat worldwide due to the global emergence of new resistance mechanisms, and limited drugs available for treatment. Multidrug-resistant bacteria like extended spectrum β-lactamase (ESBL) and carbapenemase producing members of Enterobacteriaceae (CPE), along with methicillin resistant *Staphylococcus aureus* (MRSA), and vancomycin resistant *Enterococcus* (VRE) are emerging pathogens around the globe [1]. Infections by these organisms often end with grave clinical outcomes and consume much of the health-care resources. ESBLs are the enzymes produced by bacteria that degrade extended spectrum β-lactam antibiotics such as third-generation cephalosporins, which are extensively used for the treatment of several systemic infections. The high level of expression of co-resistance to other classes of non β-lactam antibiotics by ESBL-producing pathogens is a compounding factor [2].

Gastrointestinal colonization with ESBL-producing Enterobacteriaceae (ESBL-E) may serve as an important reservoir and source of infections [2,3]. Such resistant gut flora can no longer be considered as innocent bystanders. There are several hundred specific ESBL-encoding genes. Clones harboring these genes are involved in global dissemination (http://www.lahey.org/studies/webt.asp). Most frequently found genes are *bla*_*CTX-M*_, *bla*_SHV_ and *bla*_TEM_ that are horizontally transferrable. The *bla*_CTX-M_ genes are divided into five phylogroups; CTX-M-1, CTX-M-2, CTX-M-8, CTX-M-9 and CTX-M-25 [3, 4]. The CTX-M-1 phylogroup includes CTX-M-15 which is the most prevalent globally, probably evolving towards causing a global pandemic due to its carriage by members of the Enterobacteriaceae family [5].

As is well known, the intestinal tract is a hub of many multidrug resistant pathogens, especially in immunocompromised patients in many developing countries where infection control practices are often inadequate. Immunocompromisation as in case of HIV infection characteristically causes intestinal mucosal damage, weakening the epithelial barrier, exposing the host to a broad range of microbial byproducts that, in a way could augment the progress of HIV [6]. The intestinal microbiota may take advantage of an opportunity due to the weakened immune system. Thus the burden of ESBL-E and their respective antimicrobial resistance patterns in any community whether healthy or immunocompromised have a definite impact on the health standard of that section of the population.

According to recent estimates, the global prevalence of ESBL carriage among Enterobacteriaceae in healthy individuals is around 14% [7], and Asia and Africa have experienced a tremendous increase in the numbers of ESBL-producing bacteria since the last decade. The high rate of the gut carriage in Indian and Chinese population probably accounting for the largest reservoirs of ESBL genes in Asia [2, 7].

The data on the gut colonization rate of ESBL producing bacteria in HPLWH are sparse, though Wilmore SMS, et al. recently found 13.7% prevalence of ESBL-E carriage in HIV-infected children in Zimbabwe [8]. In Nepal gut colonization of ESBL-E and CPE is not well documented. This cross-sectional study was conducted to determine and compare the rate of gastrointestinal carriage of ESBL-E and CPE in the HPLWH and healthy HIV negative population in western Nepal.

## Methods

### Ethics statement

This study was approved by the Institutional Ethics Committee, Manipal Teaching Hospital, Pokhara, Nepal. Written informed consent was obtained from each individual participating voluntarily in the study.

### Study design, site, and enrollment of participants

**Fig 1:**
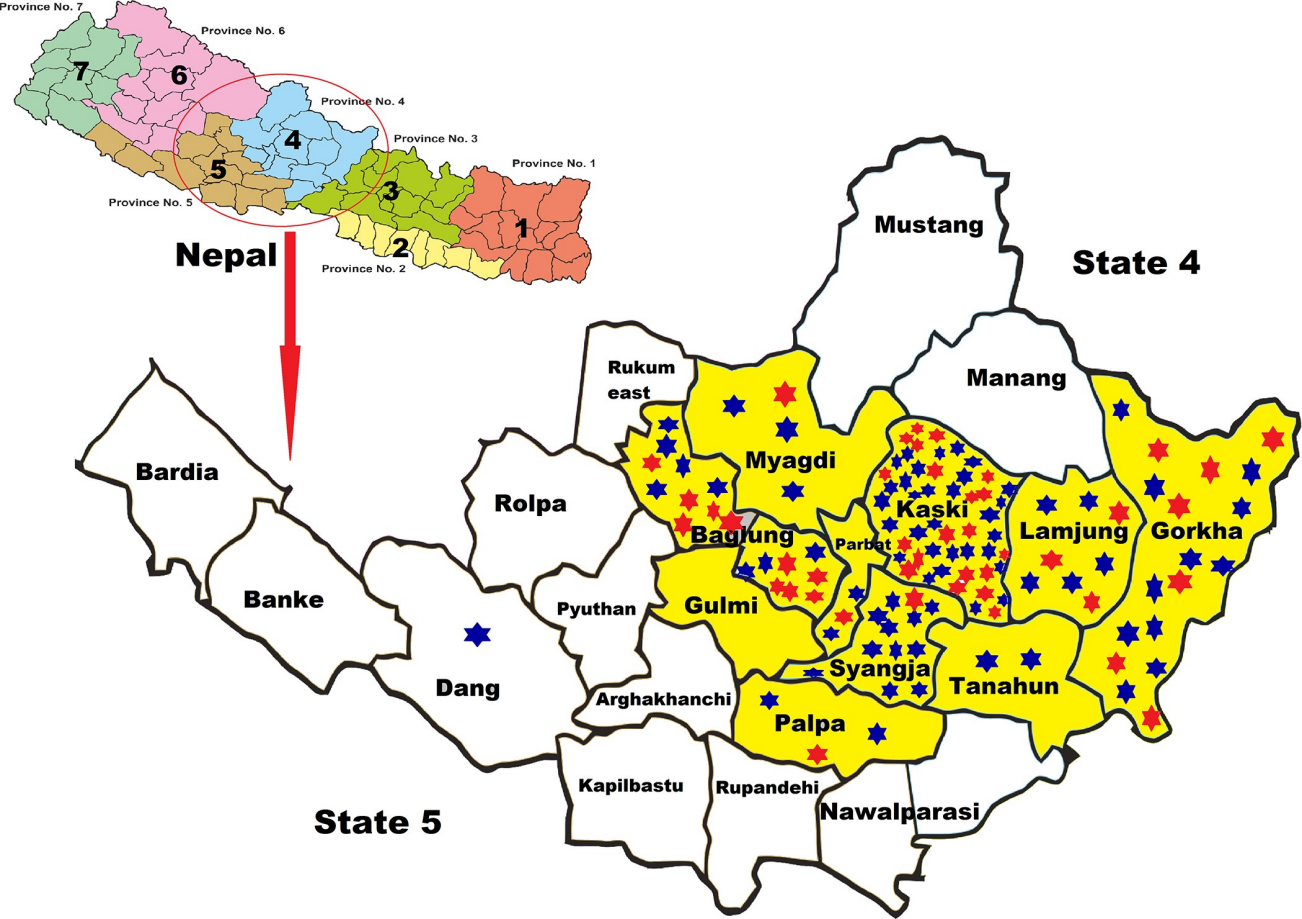
Location of 2 States (4 and 5) of Nepal where samples were collected. (The star indicates samples from HPLWH, Blue star- ESBL negative and Red star- ESBL positive. Yellow area indicates samples collected from HIV sero-negative population in the community). Map source: https://commons.wikimedia.org/wiki/Maps_of_the_world.

This cross-sectional study was conducted in nine districts of State 4 and State 5 of western Nepal. Sampling was performed between, July 2016 and January 2017. HPLWH were chosen from daycare center attendees and from those visiting Manipal Teaching Hospital and Western Regional Hospital, Pokhara for follow up. The enrolled study subjects comprised of all confirmed HIV sero-positive patients belonging to the above states (Fig 1), who volunteered to participate in the study. All participants were healthy without any signs and symptoms of infection including lymphadenopathy, fever, cachexia, unexplained diarrhea, cough, fatigue, and dizziness, as clinically evaluated by the physician, during the period of sample collection.

The specimens from HIV negative healthy volunteers from the community were obtained from the same geographical area to which HIV positive subjects belonged, using cluster random sampling method. The study area (State 4 and State 5) was divided into 23 districts; the districts were further dived to 63 municipalities and 131 rural municipalities. Out of 23 districts, samples were collected from 11 as shown in Fig 1. All municipalities or rural municipalities of 11 districts were numbered according to alphabetic order and 10 municipalities were selected by random sample calculation using software *http*:*//www*.*randomizer*.*org/form*.*htm*. On an average 32 houses were included per district and one fecal sample from one healthy volunteer per house was collected.

### Specimen collection and questionnaire

Four hundred seventy-six specimens from HPLWH and HIV negative healthy volunteers from the community were screened for ESBL and CPE. A pilot study was carried out to determine the most suitable specimen collection method. Collecting stool specimen was found to be administratively cumbersome as it involved at least two visits (once to enroll the participant and handover the specimen transport container and the second time to retrieve the collected stool specimen). Contrary to that, rectal swab could be collected along with the enrollment on the same visit and hence was the specimen of choice.

A cotton swab pre-moistened with sterile normal saline was inserted 3-5cms into the rectum and gently rotated for few seconds. The swabs were placed in a tube containing w/0.1% peptone water (HIculture Transport Swab, Himedia, India). All specimens were transported to the microbiology laboratory in specimen transport containers with ice packs. The samples were processed within a maximum of 8 hours of collection, depending upon the distance between the laboratory and the place of visit. Detailed questionnaires (S1 Questionnaires) including those delineating demographic and clinical information were included. Persons on antibiotic therapy or with a recent history of antibiotic use and/or hospitalization 30 days prior to specimen collection were excluded.

### Isolation and screening for ESBL-producing enterobacteriaceae

The rectal swab was placed in 1 ml of sterile 0.9% saline and then vortexed for 30 seconds. A small amount (10 μl) of the suspension was inoculated on commercial ESBL-selective chromogenic medium (HiMedia, India). Plates were incubated at 37°C under aerobic conditions and examined after 24 hours. The color of the colonies led to presumptive identification according to the reference color chart provided by the manufacturer. The identification was confirmed by standard biochemical tests for their speciation [9]. Screening for CPE was done on MacConkey agar with 1 μg/ml imipenem (HiMedia, India). All ESBL positive isolates were preserved in brain heart infusion-glycerol broth in stock vials and stored at -20°C for further study.

### ESBL detection by phenotypic methodsThe double-disc synergy test

Strains grown on ESBL HiChrome agar were tested for ESBL production by the Double Disc Synergy Test (DDST) using Ceftazidime (30 μg) and ceftazidime + clavulanic acid (30 μg/10 μg) [10]. Supplies were obtained from Hi-Media Laboratories Pvt. Limited, Mumbai, India.

#### AmpC detection

All the ESBL positive isolates resistant to cefoxitin in initial screening were confirmed for the AmpC enzyme co-production by two different methods (saline disc method and inhibitor-based tests) [11, 12]. Isolates which gave interpretably positive results by both the methods were considered as AmpC producers. *Klebsiella pneumoniae* ATCC 700603 and *E*. *coli* ATCC 25922 standard strains were used as positive and negative controls respectively.

#### Antimicrobial susceptibility testing

Antibiotic susceptibility testing was performed by Kirby-Bauer disk diffusion method as per CLSI guidelines [10], using eight routinely used antibiotics against the common pathogens encountered in day to day clinical practices, (tetracycline (30μg), tigecycline (15μg), amoxicillin/clavulanic acid (30μg), cefoxitin (30μg), imipenem (10μg), ciprofloxacin (30μg), amikacin (30μg), and nitrofurantoin (300μg). *Escherichia coli* (ATCC 25922) and *Klebsiella pneumoniae* (ATCC 700603) were used as the quality control strains. Isolates either resistant or intermediately sensitive to drugs were categorized as non-susceptible. For tigecycline zone size ≥18mm was considered as susceptible.

### Genotypic screening of ESBL phenotypes

Isolates displaying an ESBL phenotype were screened for ESBL encoding genes by multiplex PCR. The presence of β-lactamase genes *bla*_TEM_/*bla*_SHV_/*bla*_OXA-1_ and *bla*_CTX-M_ including phylogenetic groups 1, 2 and 9 were detected by two multiplex PCR assays [13]. Isolates positive for phylo-group1 were further characterized for CTX-M 15 gene by singleplex PCR [14].DNA isolation was performed by commercially available DNA purification kit (HipurAbacterial genomic DNA purification Kit, Himedia, India). PCR reactions were carried out in 25 μl volumes. This included 2 X Amplicon III Red Taq master mixes consisting of Tris HCL pH 8.5, (NH4)_2_ SO_4_, 4 mM MgCl_2_, 0.2% Tween 20, 0.4 mM dNTPs, and 0.2 units/μl amplicon Taq DNA polymerase. The primers were used between 0.2μM to 0.4 μM concentrations. The template was used in 1μl volume. The primers and the size of the expected DNA products for each enzyme group as previously described are listed in supplement file: S1 Table.

### Statistical analysis

Prevalence of ESBL producing isolates along with the corresponding 95% CI stratified by age group is presented by calculating frequencies and percentages. Chi-square test was used with appropriate correction for the observation. Binary logistic regression analysis was used to identify risk factors for colonization by ESBL-E. The results were presented as OR with 95% CI. Significance was set at p<0.05. Statistical analyses were performed using SPSS Statistics (IBM Corporation, Somers, NY) software, version 22.0. p< 0.05 were considered to be statistically significant.

## Results

### Demographics and clinical parameters

Specimens were collected from 119 HPLWH (82 females and 37 males). The median age of the subjects was 31 (inter-quartile range (IQR 14–43) years. The median duration of antiretroviral therapy (ART) was 3 years (IQR 2–4). Specimen from 357 healthy volunteers (213 female and 144 male) from the community with median age 28 (inter-quartile range (IQR 9–53) years were also obtained.

### ESBL-E carriage rate in HPLWH and healthy participant

ESBL-E were isolated in 45 (37.82% [CI 29.09, 47.16]) and 246 (68.91% [CI63.93, 73.49]) HPLWH and healthy participant respectively. One HPLWH yielded two ESBL isolates (*E coli* and *Klebsiella*). HPLWH had lower fecal ESBL carriage rate (odds ratio 0.274 [CI 0.178, 0.423]) compared to healthy subject’s (p<0.01). Among healthy subjects, higher ESBL carriage was observed in those who had close livestock contact and had easy access to over-the-counter (OTC) medicines (both prescription and non prescription drugs including antibiotics: oral or parenteral), compared with those who did not have close contact with livestock and no history of medication OTC{(73.3% versus 26.7%, P = 0.001) and (74.7 versus 25.3%, p = 0.001), respectively; Table 1}.Study subjects with varying demographic and related factors, such as ART duration, CD_4_ count, occupation, hospital admission, international travel, food habit, history of recent antibiotic use did not obviate differing ESBL-E colonization rates.

**Table 1:** Demographic and clinical characteristics in PLWH and healthy subjects.

	PLWH			Healthy subjects		
	ESBL positive (%)	ESBL negative (%)	p-value	ESBL positive (%)	ESBL negative (%)	p-value
**Total participants (n = 119+357 = 476)**	45 (37.82)	74(63)	-	246 (68.9)	111(31.1)	**-**
**Gender**			0.219			0.518
Male	17(45.9)	20(54.1)		102(70.8)	42(29.2)	
Female	28(34.1)	54(65.9)		144(67.6)	69(32.4)	
**Age**			0.620			0.751
</ = 18	15(34.9)	28(65.1)		113(68.1)	53(31.9)	
>18	30(39.5)	46(60.5)		133(69.6)	58(30.4)	
Median Age (IQR) years	33[14.5,43.5]	28[14,42]	-	31[9,50]	28[8,55]	-
**ART**			0.852	NA	NA	NA
Yes	39(37.5)	65(62.5)				
No	6(40)	09(60)				
**Duration of ART**			0.270	NA	NA	NA
**< = 18**	11(47.8)	12(52.2)				
**>18**	34(35.4)	62(64.6)				
Median duration of ART (IQR) years	3[1.5, 4.5]	3[2,4]				
**Occupation**			0.75			**0.03**
Agriculturist	21 (38.9)	33 (61.1)		120 (71.4)	48 (28.6)	
Study	15 (40.5)	22 (59.5)		86 (76.8)	26 (23.2)	
Business/job	06 (30)	14 (70)		24 (63.2)	14(36.8)	
Others	02 (25)	06 (75)		18 (51.4)	17 (48.6)	
**Clinical Characteristics** Admitted to hospital in last 6 months			0.063	NA	NA	NA
Yes	6(66.7)	3(33.3)				
No	39(35.5)	71(64.5)				
**History of Antibiotic use (6 months)**			0.917	NA	NA	NA
Yes	4((36.4)	7(63.6)				
No	41(38)	67(62)				
**CD4 count (cells/ /mm3)**			0.816	NA	NA	NA
<350	2(33.3)	4(66.7)				
>/ = 350	43(38.1)	70(61.9)				
**Animal contact (domestic and livestock)**			0.762			**0.001**
Yes	25(39.1)	39(60.9)		198(73.3)	72(26.7)	
No	20(36.4)	35(63.6)		48(55.2)	39(44.8)	
**OTC medicine use**			0.055			**0.001**
Yes	30(45.5)	36(54.5)		195(74.7)	66(25.3)	
No	15 (28.3)	38(71.7)		51 (53.1)	45(46.9)	
**International travel within the last 12 months**			0.63			0.52
Yes	02(28.6)	05(71.4)		08(11.6)	05(88.4)	
No	42(37.5)	70(62.5)		240(69.8)	104(30.2)	
**Food habit**			0.84			0.53
Vegetarian	04(9%)	06(8)		18(64.3)	10(35.7)	
Non-vegetarian	40(91%)	69(92)		230(69.9)	99(30.1)	

### Antibiotic resistance pattern among ESBL-E isolates

**Fig 2:**
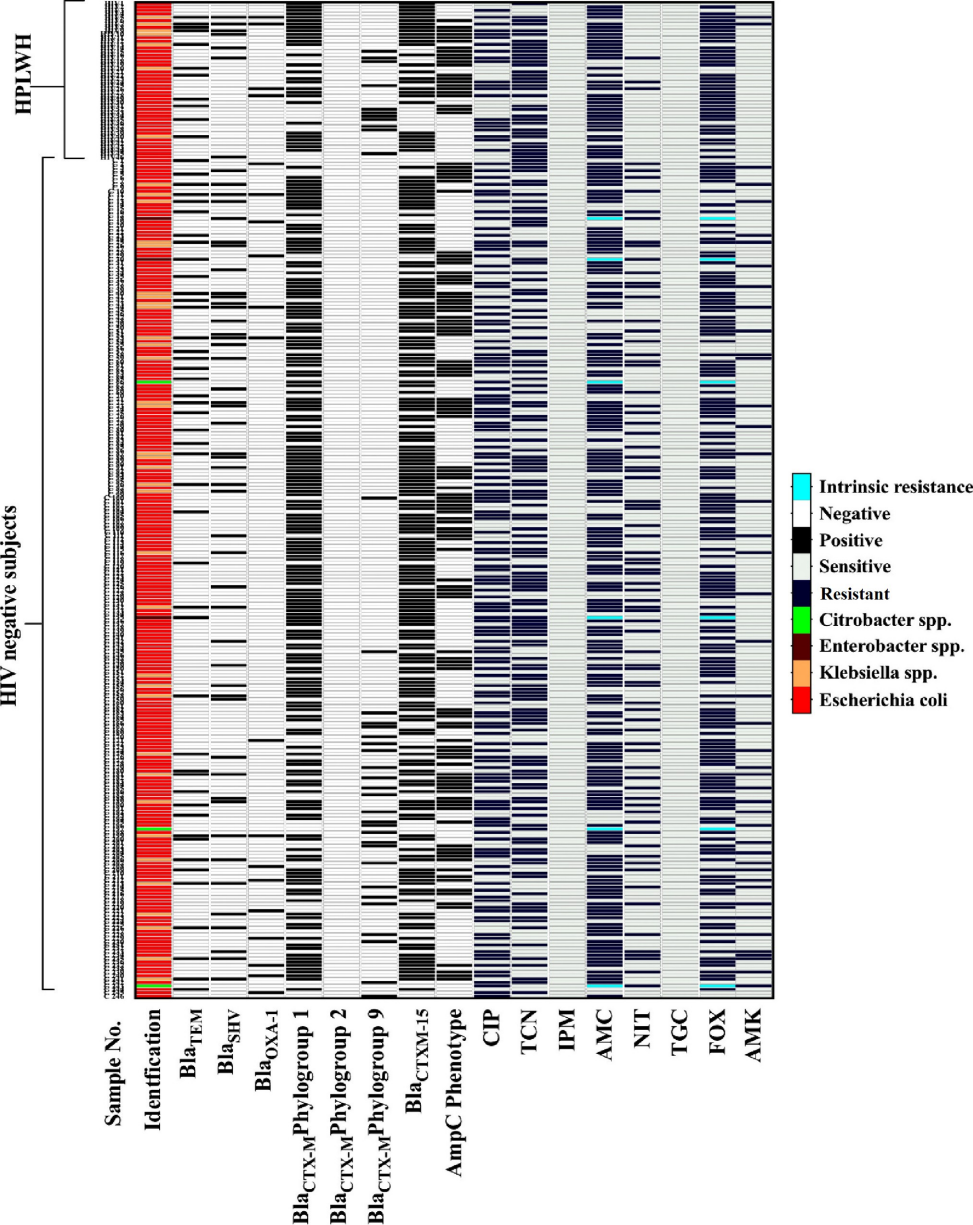
Antibiotic susceptibility and ESBL related genes identified in 292 ESBL producing isolates.

Antibiotic resistance pattern of ESBL producing bacteria isolated from HIV positive and healthy subjects are summarized in Fig 2. Of all ESBL-producing Enterobacteriaceae obtained both from HPLWH and healthy community subjects, co-resistance to amoxicillin-clavulanate was found in 80.4%, 70.7% of the isolates, to ciprofloxacin in 45.6%, 56.1% isolates, amikacin 4.3%, 17.1% isolates, tetracycline 73.9%, 52.8% isolates, cefoxitin 80.4%, 64.2% isolates, and nitrofurantoin in 6.5%, 31.3% isolates respectively. All ESBL-E isolates were, susceptible to tigecycline and imipenem.

#### AmpC and carbapenemase producers among ESBL-E isolates

AmpC co-production was observed in ESBL positive isolates in both the study groups. The co-production of ESBL and AmpC was higher in HPLWH as compared to the healthy community group (49%;22/45), and (42.2%;104/246) respectively (Table 1). In this study, no carbapenemase producing Enterobacteriaceae was isolated.

#### ESBL enzyme type prevalence

All 46 ESBL-producing Enterobacteriaceae isolated from 45 HPLWH were confirmed by PCR (Fig 2). Ten (22.2%) of these isolates showed genes coding simultaneously for multiple enzymes in various combinations while 36 (78.3%) isolates were carrying genes encoding a single enzyme. Of the 46 ESBL-producing strains, 11 (23.9%) were identified as producers of TEM-type, 4 (8.7%) of OXA-1-type and 7 (15.2%) of SHV-type β-lactamases. Among 40 (86.9%) CTX-M-producing strains, 29 (63%) and 11 (23.9%) were identified to be producing CTX-M phylogroups 1 and 9, respectively. The gene coding for the phylogroup 2 was not observed in any of the isolates. The uniplex PCR for CTX-M phylogroups-1strains revealed that 28/29 (96.6%) isolates were of blaCTX-M-15 ESBL type.

Out of 246 phenotypically confirmed ESBL-producing Enterobacteriaceae from healthy participants, 242 (98.4%) were confirmed by PCR (Fig 2). Forty-five (18.6%) of these isolates showed genes coding simultaneously for multiple enzymes in various combinations while 197 (81.4%) isolates were carrying genes coding for single type of enzyme. Of the 242 ESBL-isolates, 45 (18.6%) were positive for TEM-type, 15 (6.2%) as OXA-1-type and 47(19.4%) as SHV type β-lactamases. Among 200 (82.6%) CTX-M-producing strains, 174(87%), and 26(13%) isolates belonged to CTX-M phylogroups 1 and 9 respectively. All of 174(100%) CTX-M phylogroups 1 were identified as the blaCTX-M-15. The gene coding for the phylogroup 2 was not observed in any of the isolates. The dominant species among ESBL-E from both HPLWH and healthy participant groups were *E*. *coli* (37/46, 80.4%; 206/246, 83.7%), followed by *K*. *pneumoniae* (6/46, 13%; 32/246, 13%). In addition, *Enterobacter* species (1/46, 2.2%; 3/246, 1.2%), *K*. *oxytoca* (2/46, 4.4%; 2/246, 0.8%), and *Citrobacter* spp. (0/46, 00%; 3/246, 1.2%), were found among the ESBL producers.

### Predictors of ESBL carriage

In this study, univariate and multivariate logistic regression model was used to analyze risk factors associated with ESBL colonization (Table 2). Among the HPLWH the odd of ESBL carriage was 1.537 times (95% CI 0.349, 6.762) higher in those on ART than in those without ART. Among the HPLWH taking antibiotics, there was a 21% [0.787 (95% CI 0.179, 3.464)] odds reduction of ESBL carriage rate. In addition, the odd of ESBL carriage in HPLWH were 3.638 times (0.793, 16.68) higher in those admitted to hospital in the previous six months.

**Table 2:** Univariate and multivariate analysis of factors associated with ESBL-E carriage.

	PLWH(n = 119)				Healthy subjects (n = 357)			
	Univariate OR (95% CI)	p-value	Adjusted multivariate OR (95% CI)	Final Adjusted multivariate OR (95% CI)	Univariate OR (95% CI)	p-value	Adjusted multivariate OR (95% CI)	Final Adjusted multivariate OR
**Gender**		0.221				0.518		
**Male**	1.639 (0.743, 3.617)		1.867(0.796, 4.380)		1.164(0.743, 1.843)		2.244(1.392, 4.511)	
**Female**	1		1		1		1	
**Age**		0.620				0.751		
**< = 18**	1		1		1		1	
**>18**	1.217(0.559, 2.650)		1.281(0.515, 3.186)		1.076(0.686, 1.685)		1.091(0.682, 1.746)	
**ART**		0.852			NA	NA	NA	NA
Yes	0.9 (0.298, 2.722)		1.535(0.334, 7.047)	1.537(0.349, 6.762)				
No	1		1	1				
**ART duration**		0.273			NA	NA	NA	NA
< = 1	1		1	1				
>1	0.598(0.239, 1.499)		0.432(0.124, 1.505)	0.473(0.139, 1.614)				
**Clinical Characteristics Admitted to hospital in last 6 months**		0.79			NA	NA	NA	NA
**Yes**	3.641 (0.863, 15.366)		3.414(0.740, 15.750	3.638(0.793, 16.68)				
**No**	1		1	1				
**History of Antibiotic use (6 months)**		0.917			NA	NA	NA	NA
**Yes**	0.934(0.257, 3.387)		0.703(0.156, 3.159)	0.787(0.179, 3.464)				
**No**	1		1	1				
**CD4 count (cells/mm**^**3**^ **)**		0.817			NA	NA	NA	NA
**<350**	0.814(0.143, 4.634)		1.008 (0.163, 6.237)	0.956(0.158, 5.785)				
**>/ = 350**	1		1	1				
**Animal contact (domestic and livestock)**		0.762				0.002		
**Yes**	1.122(0.533, 2.361)		1.073(0.447, 2.571)	1.153(0.515, 2.581)	2.234(1.353, 3.689)		2.244(1.301, 3.871)	1.866(1.109, 3.140)
**No**	1		1	1	1		1	1
**OTC**		0.050				0.001		
**Yes**	2.111(0.978, 4.557)		2.108(0.937, 4.740)	1.988(0.898, 4.401)	2.607(1.600, 4.249)		3.528(1.949, 6.388)	2.296(1.389, 3796)
**No**	1		1	1	1		1	1

We found significant rate of ESBL colonization among individuals who had close contact with livestock [HPLWH study group OR 1.153 (95% CI 0.515, 2.581) as compared to healthy HIV negative group 1.866 (95%CI 1.109, 3.140) and also among individuals using medication over-the-counter [HPLWH OR 1.988 (95%CI 0.898, 4.401) vs. the healthy HIV negative subjects 2.296 (95% CI 1.389, 3796). Majority of the participants were the frequent consumer of non-vegetarian diet and had no previous international travel history, therefore these variables were not included in the analysis. There was no significant association between ESBL-E carriage and gender, age, ART duration, antibiotic use, or CD4 count.

## Discussion

Prevalence of ESBL-carrying Enterobacteriaceae in the gut of healthy people has been reported globally, but there is little information about such prevalence and the risk factors thereof, among the HPLWH. Surveillance for gut colonization of ESBL-producing bacteria in humans provides information about the fecal flora which may contaminate the products entering the food chain. Limited availability of antibiotics for treating patients infected by ESBL and CPE producing bacteria represents a great concern for healthcare.

According to Woerther PL et al. [2] and the WHO 2010 population census (http://www.who.int/research), the number of ESBL-E carriers was estimated over 1.1 billion in Southeast Asia alone. The Western Pacific and Eastern Mediterranean regions rank second and third, with 280 and 180 million carriers, respectively, ahead of Africa, where 110 million carriers are estimated to be present. America and Europe appear to be far behind, with 48 and 35 million immunocompetent carriers, respectively. Few studies from Nepal reported that ESBL prevalence among clinical Enterobacteriaceae isolates ranged between 13 to 39% with CTX-M-15 being the predominant genotype [15–17]. Shrestha B, et al.found15% prevalence of carbapenemase-producing hospital strains of *E coli* encoding various NDM-Type-Metallo-β-Lactamases [18], but studies pertaining to gastrointestinal colonization rate and genotypic distribution of ESBL-E in either hospitalized patients or healthy subjects in Nepal are lacking. Healthy individuals at the community level are an important reservoir for ESBLs and the global report shows that colonization rate appears to be increasing over time (approx. >5% annually) [2, 7]. The present study demonstrated a high rate (68.9%) of gut colonization by ESBL-E in healthy subjects from the community in western Nepal. Colonization rate seems to be quite higher in this geographical area compared to previous studies around the globe [1, 2, 15, 17]. Rates reported in a systematic review and meta-analysis by S Karanika, et al. in 2016 [7], showed that the global pooled prevalence of ESBL class A colonization was 14% (95% CI, 9, 20). The pooled prevalence was higher in Asia and Africa (ranging from 46%, (95% CI, 29, 63) to 15%, (95% CI, 4, 31) and lower but still significant in central (3%, 95% CI, 1, 5), northern (4%, 95% CI, 2, 6), and southern Europe (6%, 95% CI, 1, 12) and the Americas (2%, 95% CI, 0, 5).

Surprisingly, HPLWH in the present study showed low colonization rates of ESBL-E as compared to healthy subjects (37.8% v/s 68.9%). Padmavathy K, et al. while working on the virulence of uropathogenic *E coli* observed that ESBL producers were more common among HIVsero-negative patients compared to the HIV sero-positive study subjects (16.7% vs 3.9%, P = 0.04407).Contrary to this, multiple antibiotic resistance (ESBL+ AmpC+ fluoroquinolone) in her study, was significantly higher among the isolates from HIV positive patients compared to the HIV negative cohort (59.2% vs 19.0%, P = 0.000036) [19]. Much in agreement with the present study, Karol JM, et al. found a low rate of ESBL-E among the clinical isolates in HIV positive patients (p = 0.92, OR 1:1.04) [20].

Though, various studies in the past reported low ESBL carriage among the clinical isolates in HIV positive individuals, none of these studies have highlighted these aspects about the gut colonizers [8, 19, 20]. The exact modus operandi for such low colonization rate in HIV infected subjects is still conjectural. However, HIV patients are more prone to infections, and hence are more likely to be hospitalized and consume antimicrobial agents than HIV negative persons; a proposition that could support the view that HIV status might not always be a well-established risk factor for ESBL carriage. Thus further studies will be required to draw a meaningful interpretation.

Most of the HPLWH among our study subjects were on ART. Studies related to any association between gut colonization with MDR/ESBL producing Enterobacteriaceae and ART are lacking. However, occasional report stating the emergence of MDR *Mycobacterium tuberculosis* among HIV patients on ART [21] may not support the fascinating and novel observation of low gut colonization rate in HPLWH/HIV group in our study, but could, in a way, support the finding of the emergence of drug resistance among the isolates as shown in our study. However, an *in vivo* study conducted earlier, using a mouse model of experimental influenza stated that treatment with antiviral agents improved survival in mice from 0% to 75%, diminishing the occurrence of bacterial colonization, thereby reducing secondary bacterial pneumonia by a yet unknown mechanism [22].These findings necessitate further studies to find any link between ART and development of antibiotic resistance vis-a-vis low *in vivo* colonization rate

Considering the risk factors for ESBL-E colonization, it has well been established that gut microbiota are severely impacted and the altered bacterial communities play vital roles, directly or indirectly in disease progression. Previous studies [7] identified various risk factors for ESBL-E gut colonization such as; age above 60 years, various co-morbidity conditions, antibiotic use for the prior 4 or 12 months and international travel. In this study, we found close livestock contact and over-the-counter medication (involves both prescription and non-prescription drugs) among healthy subjects as the major risk factors for ESBL colonization. In Nepal and many other neighboring developing countries, people have easy access to all kinds of antibiotics over the counter at pharmacies and private retail shops without any prescription by a registered medical practitioner, which could lead to a calamitous antibiotic resistance situation. The Government of Nepal has designed the National Antibiotic Treatment Guidelines in 2014 [23], which is still in course to be implemented. Dispensing practices of antibiotics in community pharmacies was recently studied in central Nepal by Ansari M [24], who found that cephalosporins (69.8% cases) were the most frequently dispensed antibiotics, followed by penicillins (68.3%), macrolides (57.1%), fluoroquinolones (49.1%) and sulfonamides (19.3%). Besides, the practice of dispensing antibiotics without prescription and by non-pharmacists were found to be very common.

Recently the most important antibiotic resistance problems identified by the Alliance for the Prudent Use of Antibiotics (APUA) Nepal members (https://apua.org/nepal/), included lack of regulation in human, animal and agricultural antibiotic use, justifying the formulation of optimum treatment guidelines along with strict enforcement of 'prescription-only' laws in the country.

We did not find any significant risk involved with gender, age, ART duration, CD4 count, food habit or international travel. The small sample size in this study could have resulted in reduced power to identify exact risk factors. A larger study may be able to unravel these riddle.

It is well known that ESBL producing isolates often have high rates of co-resistance to commonly used antimicrobials. The results of antimicrobial sensitivity testing revealed that all ESBL producers in this study often co-expressed resistance to many other classes of antibiotics. Most of the isolates from HPLWH group were susceptible to nitrofurantoin and amikacin but showed resistance to cefoxitin, tetracycline and amoxicillin/clavulanic acid. Ciprofloxacin susceptibility was quite lower (41%) in HPLWH group than in the healthy individuals (57.8%). A recent study from Tanzania showed that ESBL producing isolates had high rates of resistance to commonly used antimicrobials [25]. Previous studies [26] too documented that administration of several antibiotics posed definite risks for multidrug-resistant Enterobacteriaceae colonization. However, it would be of much interest to find out any relationship between antibiotic consumption rates and development of resistance in the gut micro biota of HPLWH and HIV negative individuals, a kind of proposition that warrants investigation in this geographical area.

Genotype prevalence depicted in this study is similar to that projected vide the global reports [2, 7, 18]. Among *bla*_*CTX-M*_ positive isolates, the most dominant genotype was *bla*_*CTX-M-15*_ (70%) and the dominant bacterial species were *E*. *coli* (83%). Our results were in agreement with previous studies on ESBL-producing Enterobacteriaceae conducted in other places in the Asian subcontinent [2]. In addition, Hal Jones C, et al. reported that 44% of the ESBL-E strains collected globally harbored the genes for multiple ESBLs. In this study, 10 (22.2%) of the isolates from HIV positive subjects and 45 (18.6%) from healthy voluntaries possessed two or more ESBL genes. Most of the isolates with multiple ESBL genes had *bla*_*CTX-M-15*_ along with other beta-lactamases, implying that *bla*_*CTX-M-15*_ had the potential to coexist with other beta-lactamases.

The observation of a high prevalence of the CTX-M 15 like genotype, in the present study, further substantiated the earlier notion [2] that CTX-Ms were the predominant enzymes among the strains during carriage. This also supported the contention that mobile genetic elements were the cornerstones of the, so-called, current CTX-M pandemic. Plasmids were involved in the intercontinental spread of CTX-M-15, and community outbreaks could be because of strain to strain transmission of plasmids [27]. The plausibility of such scenario was further supported by other similar studies from different geographical areas [28, 29] which unanimously agreed that community had a major role to play to act as the main reservoir of CTX-M type ESBL. The presence of this type of enzyme in hospitalized patients could be a secondary consequence and could give rise to subsequent nosocomial outbreaks [30, 31]. Our results not only showed that CTX-M type ESBL has spread to the community, but also that carriage rates were on the rise. This is a major public health concern, though the drivers of this catastrophic spread have not yet been fully elucidated. Future efforts should focus on determining the presence of transferable resistance plasmids through conjugation experiments and incorporating whole-genome sequencing and genomic analysis as additional confirmatory tools to track the spread of these resistance genes within the population [32].

The limitation of the present study was PCR products were not subjected to sequencing to confirm the ESBL genotypes. As we screened for the presence of ESBL genes by PCR, we are unable to further speculate whether specific ESBL family variants are stratified within and between the groups.

## Conclusion

This study identifies the low fecal carriage rate of ESBL producing bacteria in HPLWH group compared to healthy individuals in western Nepal. The factors responsible for this inverse relationship of HIV status and gut colonization by ESBL-E are unidentified and require further large-scale study. This is the first study in Nepal that has demonstrated a high gut colonization rate by ESBL-E in the community, predominantly of blaCTX-M-15 genotype. We found that livestock contact and over-the-counter medications were significantly associated with a higher ESBL-E carriage rate among healthy subjects. Implementation of strict antibiotic policy and jurisdiction over pharmacy practices are crucial in preventing such colonization.

## Supporting information

S1 Questionnaires Questionnaires for rectal swab/stool specimen collection. (DOC)

S1 Table List of primers used. (DOC)

## References

[1] Ramphal R, Ambrose PG. Extended-spectrum beta-lactamases and clinical outcomes: current data. Clin Infect Dis. 2006 4 15; 42 Suppl 4:S164–72. 16544267 10.1086/500663

[2] Woerther PL, Burdet C, Chachaty E, Andremont A. Trends in human fecal carriage of extended-spectrum β-lactamases in the community: toward the globalization of CTX-M. Clin Microbiol Rev. 2013 10;26(4):744–58. 10.1128/CMR.00023-13 24092853 PMC3811232

[3] Birkett CI, Ludlam HA, Woodford N, Brown DF, Brown NM, Roberts MT, et al Real-time TaqMan PCR for rapid detection and typing of genes encoding CTX-M extended-spectrum beta-lactamases. J Med Microbiol. 2007 1;56(Pt 1):52–5. 10.1099/jmm.0.46909-0 17172517

[4] D'Andrea MM, Arena F, Pallecchi L, Rossolini GM. CTX-M-type β-lactamases: a successful story of antibiotic resistance. Int J Med Microbiol. 2013 8;303(6–7):305–17. 10.1016/j.ijmm.2013.02.008 23490927

[5] Nicolas-Chanoine MH, Bertrand X, Madec JY. *Escherichia coli* ST131, an intriguing clonal group. Clin Microbiol Rev. 2014 7;27(3):543–74. 10.1128/CMR.00125-13 24982321 PMC4135899

[6] Nwosu FC, Avershina E, Wilson R, Rudi K. Gut Microbiota in HIV Infection: Implication for Disease Progression and Management. Gastroenterol Res Pract. 2014;2014:803185 10.1155/2014/803185 25024700 PMC4082943

[7] Karanika S, Karantanos T, Arvanitis M, Grigoras C, Mylonakis E. Fecal Colonization With Extended-spectrum Beta-lactamase-Producing Enterobacteriaceae and Risk Factors Among Healthy Individuals: A Systematic Review and Metaanalysis. Clin Infect Dis. 2016 8 1;63(3):310–8. 10.1093/cid/ciw283 27143671

[8] Wilmore SMS, Kranzer K, Williams A, Makamure B, Nhidza AF, Mayini J, et al Carriage of extended-spectrum beta-lactamase-producing Enterobacteriaceae in HIV-infected children in Zimbabwe. J Med Microbiol. 2017 5;66(5):609–615. 10.1099/jmm.0.000474 28513417 PMC5817228

[9] Winn WC. Koneman's color atlas and textbook of diagnostic microbiology. 6th ed Philadelphia: Lippincott williams & wilkins; 2006.

[10] Clinical Laboratory Standards Institute (2011) Performance standards for antimicrobial susceptibility testing: twenty-first Informational Supplement. M100–S21. CLSI, Wayne, PA.

[11] Singhal S, Mathur T, Khan S, Upadhyay DJ, Chugh S, Gaind R, et al Evaluation of methods for AmpC beta-lactamase in gram negative clinical isolates from tertiary care hospitals. Indian J Med Microbiol. 2005 4;23(2):120–4. 15928443 10.4103/0255-0857.16053

[12] Ingram PR, Inglis TJ, Vanzetti TR, Henderson BA, Harnett GB, Murray RJ. Comparison of methods for AmpC β-lactamase detection in Enterobacteriaceae. J Med Microbiol. 2011 6;60(Pt 6):715–21. 10.1099/jmm.0.029140-0 21372181

[13] Dallenne C, Da Costa A, Decré D, Favier C, Arlet G. Development of a set of multiplex PCR assays for the detection of genes encoding important beta-lactamases in Enterobacteriaceae. J Antimicrob Chemother. 2010 3;65(3):490–5. 10.1093/jac/dkp498 20071363

[14] Çakir Erdoğan D, Cömert F, Aktaş E, Köktürk F, Külah C. Fecal carriage of extended-spectrum beta-lactamase-producing Escherichia coli and *Klebsiella* spp. in a Turkish community. Turk J Med Sci. 2017 2 27;47(1):172–179. 10.3906/sag-1512-9 28263486

[15] Pokhrel RH, Thapa B, Kafle R, Shah PK, Tribuddharat C. Co-existence of beta-lactamases in clinical isolates of *Escherichia coli* from Kathmandu, Nepal. BMC Res Notes. 2014 10 7;7:694 10.1186/1756-0500-7-694 25287013 PMC4197279

[16] Chander A, Shrestha CD. Prevalence of extended spectrum beta lactamase producing *Escherichia coli* and *Klebsiella pneumoniae* urinary isolates in a tertiary care hospital in Kathmandu, Nepal. BMC Res Notes. 2013 11 25;6:487 10.1186/1756-0500-6-487 24274894 PMC4222089

[17] Sherchan JB, Hayakawa K, Miyoshi-Akiyama T, Ohmagari N, Kirikae T, et al Clinical epidemiology and molecular analysis of extended-spectrum-β-lactamase-producing Escherichia coli in Nepal: characteristics of sequence types 131 and 648. Antimicrob Agents Chemother. 2015;59(6):3424–32. 10.1128/AAC.00270-15 25824221 PMC4432170

[18] Shrestha B, Tada T, Shimada K, Shrestha S, Ohara H, Pokhrel BM, et al Emergence of Various NDM-Type-Metallo-β-Lactamase-Producing *Escherichia coli* Clinical Isolates in Nepal. Antimicrob Agents Chemother. 2017 11 22;61(12). pii: e01425–17. 10.1128/AAC.01425-17 28993336 PMC5700343

[19] Padmavathy K, Padma K, Rajasekaran S. Extended-spectrum β-lactamase/AmpC-producing uropathogenic *Escherichia coli* from HIV patients: do they have a low virulence score? J Med Microbiol. 2013 3;62(Pt 3):345–51. 10.1099/jmm.0.050013-0 23161767

[20] Marwa KJ, Mushi MF, Konje E, Alele PE, Kidola J, Mirambo MM. Resistance to Cotrimoxazole and Other Antimicrobials among Isolates from HIV/AIDS and Non-HIV/AIDS Patients at Bugando Medical Centre, Mwanza, Tanzania. AIDS Res Treat. 2015;2015:103874 10.1155/2015/103874 25793123 PMC4352486

[21] Iseman MD. Treatment of multidrug-resistant tuberculosis. N Engl J Med. 1993 9 9;329(11):784–91. Review. Erratum in: N Engl J Med 1993 Nov 4;329(19):1435. 10.1056/NEJM199309093291108 8350889

[22] McCullers JA. Effect of antiviral treatment on the outcome of secondary bacterial pneumonia after influenza. J Infect Dis. 2004 8 1;190(3):519–26. 10.1086/421525 15243927

[23] National antibiotic treatment guidelines. Ministry of Health and Population, Government of Nepal, Kathmandu, Nepal. Available from: http://www.mohp.gov.np/images/pdf/guideline/National_Antibiotic_Treatment_Guidelines.pdf

[24] Ansari M. Evaluation of community pharmacies regarding dispensing practices of antibiotics in two districts of central Nepal. PLoS One. 2017 9 26;12(9):e0183907 10.1371/journal.pone.0183907 28949990 PMC5614431

[25] Tellevik MG, Blomberg B, Kommedal Ø, Maselle SY, Langeland N, Moyo SJ. High Prevalence of Faecal Carriage of ESBL-Producing Enterobacteriaceae among Children in Dar es Salaam, Tanzania. PLoS One. 2016 12 9;11(12):e0168024 10.1371/journal.pone.0168024 27936054 PMC5148075

[26] Gardam MA, Burrows LL, Kus JV, Brunton J, Low DE, Conly JM, et al Is surveillance for multidrug-resistant enterobacteriaceae an effective infection control strategy in the absence of an outbreak? J Infect Dis. 2002 12 15;186(12):1754–60. 10.1086/345921 12447761

[27] Peirano G, Mulvey GL, Armstrong GD, Pitout JD. Virulence potential and adherence properties of *Escherichia coli* that produce CTX-M and NDM β-lactamases. J Med Microbiol. 2013 4;62(Pt 4):525–30. 10.1099/jmm.0.048983-0 23319311

[28] Johnson JR, Porter SB, Zhanel G, Kuskowski MA, Denamur E. Virulence of *Escherichia coli* clinical isolates in a murine sepsis model in relation to sequence type ST131 status, fluoroquinolone resistance, and virulence genotype. Infect Immun. 2012 4;80(4):1554–62. 10.1128/IAI.06388-11 22311928 PMC3318413

[29] Escobar-Páramo P, Grenet K, Le Menac'h A, Rode L, Salgado E, Amorin C, et al Large-scale population structure of human commensal *Escherichia coli* isolates. Appl Environ Microbiol. 2004 9;70(9):5698–700. 10.1128/AEM.70.9.5698-5700.2004 15345464 PMC520916

[30] Naseer U, Olsson-Liljequist BE, Woodford N, Dhanji H, Cantón R, Sundsfjord A, et al Multi-locus variable number of tandem repeat analysis for rapid and accurate typing of virulent multidrug resistant Escherichia coli clones. PLoS One. 2012;7(7):e41232 10.1371/journal.pone.0041232 22859970 PMC3407997

[31] Reyna-Flores F, Barrios H, Garza-Ramos U, Sánchez-Pérez A, Rojas-Moreno T, Uribe-Salas FJ, et al Molecular epidemiology of *Escherichia coli* O25b-ST131 isolates causing community-acquired UTIs in Mexico. Diagn Microbiol Infect Dis. 2013 7;76(3):396–8. 10.1016/j.diagmicrobio.2013.03.026 23774006

[32] Orlek A, Stoesser N, Anjum MF, Doumith M, Ellington MJ, Peto T, et al Plasmid Classification in an Era of Whole-Genome Sequencing: Application in Studies of Antibiotic Resistance Epidemiology. Front Microbiol. 2017 2 9;8:182 10.3389/fmicb.2017.00182 28232822 PMC5299020

