# Supplementary material for: Low rate of gut colonization by extended-spectrum β-lactamase producing Enterobacteriaceae in HIV infected persons as compared to healthy individuals in Nepal
Source: PLoS One. 2019 Feb 19;14(2):e0212042. doi: 10.1371/journal.pone.0212042 (PMC6380550; doi:10.1371/journal.pone.0212042)
Supplement: S1 Questionnaires — (DOC) [file pone.0212042.s001.doc]

1. **Questionnaires for rectal swab/stool specimen collection in community**

**Sl. No: VDC No.: House no.: Participant name and Address:**

| **Total members in family** | **History of infection & antibiotic use** | **OTC medication history**  **(if yes-specify type and duration)** | **History of Hospitalization** | **Animal contact**  **(type, duration, and animal breed)** | **Close contact with health care person** | **International Travel history** |  |
| --- | --- | --- | --- | --- | --- | --- | --- |
| **Adult male**  1.Name :  2. Age:  3. Occupation:  4. Diet: Veg/Non-veg |  |  |  |  |  |  |  |
| **Adult Female**  1.Name:  2. Age:  3. Occupation:  4. Diet: veg/non-veg |  |  |  |  |  |  |  |
| **Children:**  1.Name:  2. Age/gender:  3. Occupation:  4. Diet: veg/non-veg |  |  |  |  |  |  |  |

**Remarks:**

**Signature of field work candidate:**

| **To be filled by Research investigator:**   1. Total no. of specimens received : 2. Date and time: 3. Remarks: 4. Signature: |
| --- |

**2. Questionnaires for rectal swab collection: Retro-positive cases**

**Sl. No: VDC NO.: ART center Name:**

**Participant name and address: Physician remarks:**

| **Total members in family** | **History of infection & antibiotic use (in last 6months)** | **OTC medication history**  **(if yes-specify type and duration)** | **History of Hospitalization in last 6 months** | **Animal contact (type, duration, and animal breed)** | **Close contact with health care person** | **ART duration** | **CD4 count** | **Travel History** |
| --- | --- | --- | --- | --- | --- | --- | --- | --- |
| **Adult male**  1.Name :  2. Age:  3. Occupation:  4. Diet: veg / non-veg |  |  |  |  |  |  |  |  |
| **Adult female**  1.Name:  2. Age:  3. Occupation:  4. Diet: veg / non-veg |  |  |  |  |  |  |  |  |
| **Children:**  1.Name:  2. Age/gender:  3. Occupation:  4. Diet: veg / non-veg |  |  |  |  |  |  |  |  |

**Remarks:**

**Signature of field work candidate:**

| **To be filled by Research investigator:**  1. Total no. of specimens received :   1. Date and time: 2. Remarks: 3. Signature: |
| --- |
