# Supplementary material for: Low rate of gut colonization by extended-spectrum β-lactamase producing Enterobacteriaceae in HIV infected persons as compared to healthy individuals in Nepal
Source: PLoS One. 2019 Feb 19;14(2):e0212042. doi: 10.1371/journal.pone.0212042 (PMC6380550; doi:10.1371/journal.pone.0212042)
Supplement: S1 Table — (DOC) [file pone.0212042.s002.doc]

**S1Table : List of primers used**

| **Sl. no** | **PCR name** | **β-lactamase(s) targeted** | **Sequence (50 –30)** | **Length**  **(bases)** | **Amplicon**  **size (bp)** | **Primer**  **concentration**  **(pmol/mL)** | **Reference** |
| --- | --- | --- | --- | --- | --- | --- | --- |
| **1** | **Multiplex I**  TEM, SHV and  OXA-1-like | **TEM variants** including TEM-1 and  TEM-2 | F-CATTTCCGTGTCGCCCTTATTC  R-CGTTCATCCATAGTTGCCTGAC | 22  22 | 800 | 0.4  0.4 | 13 |
|  |  | **SHV variants** including SHV-1 | F-AGCCGCTTGAGCAAATTAAAC  R-ATCCCGCAGATAAATCACCAC | 21  21 | 713 | 0.4  0.4 |  |
|  |  | **OXA-1, OXA-4 and OXA-30** | F-GGCACCAGATTCAACTTTCAAG  R-GACCCCAAGTTTCCTGTAAGTG | 22  22 | 564 | 0.4  0.2 |  |
| 2 | **Multiplex I I**  CTX-M group 1,  group 2 and group 9 | **Variants of CTX-M group 1** including CTX-M-1, CTX-M-3 and CTX-M-15 | F-TTAGGAARTGTGCCGCTGYAb  R-CGATATCGTTGGTGGTRCCAT^b^ | 20  21 | 688 | 0.2  0.2 | 13 |
|  |  | **Variants of CTX-M group 2** including CTX-M-2 | F-CGTTAACGGCACGATGAC  R-CGATATCGTTGGTGGTRCCAT^b^ | 18  21 | 404 | 0.4  0.4 |  |
|  |  | **Variants of CTX-M group 9 including CTX-M-9 and CTX-M-14** | F-TCAAGCCTGCCGATCTGGT  R-TGATTCTCGCCGCTGAAG | 19  18 | 561 |  |  |
| 3 | **Singleplex** | **CTX-M-15** | F-CACACGTGGAATTTAGGGACT  R-GCCGTCTAAGGCGATAAACA |  | 996 |  | 14 |

**Legend**: **^b^Y**=T or C; **R**=A or G.
